# Hot-Air Spinning Technology Enables the High-Efficiency Production of Nanofiber

**DOI:** 10.3390/nano15080578

**Published:** 2025-04-11

**Authors:** Guo-Dong Zhang, Yuan Gao, Pi-Hang Yu, Chao Zhang, Chuan-Hui Guo, Seeram Ramakrishna, Yun-Ze Long, Jun Zhang

**Affiliations:** 1 Shandong Key Laboratory of Medical and Health Textile Materials, Collaborative Innovation Center for Nanomaterials & Devices, College of Physics, Qingdao University, Qingdao 266071, China; 2018205782@qdu.edu.cn (G.-D.Z.); 2021010006@qdu.edu.cn (Y.G.); yupihang1@qdu.edu.cn (P.-H.Y.); 2022020307@qdu.edu.cn (C.Z.); guochuanhui@qdu.edu.cn (C.-H.G.); 2 Center for Nanofibers & Nanotechnology, Department of Mechanical Engineering, National University of Singapore, Singapore 117574, Singapore; 2018205649@qdu.edu.cn; 3 State Key Laboratory of Bio-Fibers & Eco-Textiles, Qingdao 266071, China

**Keywords:** solution blow spinning, water-soluble polymer, nanofiber materials

## Abstract

Water is the most environmentally friendly solvent; however, conventional solution spinning using water as a solvent is challenging due to its low evaporation rate. We developed a double-pronged solution blow spinning (DP-SBS) system. This spinning technique significantly enhances solvent evaporation, and the designed structure (double-pronged) avoids the common problem of needle clogging caused by heating. DP-SBS enables high-yield production of water-soluble polymer nanofibers, with a production rate of up to 5.94 g/h, which far exceeds what can be achieved with traditional electrospinning or solution blow spinning. This method is also highly efficient for producing non-water-soluble polymer nanofibers, achieving a production rate of up to 7.91 g/h, the highest reported value to date. Additionally, this approach can be used to produce not only common two-dimensional fiber membranes but also fiber sponges in a single step using the double-pronged airflow system. For the first time, chitosan nanofiber sponges were successfully produced and demonstrated to have excellent hemostatic properties in medical hemostasis. This method can also be extended to the production of other 3D nanomaterials, such as mullite nanofiber sponges, which exhibit outstanding thermal insulation performance at high temperatures.

## 1. Introduction

Nanofibers, as an advanced material, possess excellent mechanical properties, an extremely high specific surface area, and high porosity [1,2,3,4]. They show a wide range of application prospects in energy production, water treatment, environmental remediation, biomedicine, flexible electronics, and aerospace [5,6,7,8,9,10,11,12,13,14]. However, the large-scale production of nanofibers still faces several challenges, including solvent selection, production rate, and quality control. Among them, the spinning method with green solvents—such as water—often encounters problems of adhesion of the polymer jet and droplet residue due to incomplete solvent volatilization. Traditional spinning methods struggle to address the problems arising from the slow evaporation rate of solvents. Therefore, to meet the growing market demand for nanofiber products and to advance research and development in various fields, there is an urgent need for a preparation method that is green, highly efficient, simple, and safe.

Currently, various nanofiber preparation techniques have been reported but some—like template synthesis, molecular self-assembly, and the hydrothermal method—are only suitable for laboratory-scale research and are challenging to apply in large-scale industrial production [15,16,17]. While melt-blow spinning has been industrialized, it produces fibers with a larger diameter (>1 μm) and can only produce thermoplastic polymer fibers [18,19]. The primary methods currently used to prepare nanofibers include electrospinning (ES) and solution blow spinning (SBS). Due to its versatility, simplicity, and low cost, ES is widely used to prepare nanofibers across various fields. Moreover, solvent evaporation rates in electrospinning methods can be easily controlled by adjusting environmental conditions and process parameters. However, it requires high-voltage electricity, posing significant safety risks. Additionally, the dielectric constant required for solvents in electrospinning limits the spinning of high-salt-concentration solutions [20,21,22]. SBS utilizes high-speed airflow to create shear force at the gas–liquid interface. When the shear force exceeds the liquid’s surface tension, liquid jets are generated, which then undergo stretching by the airflow and evaporation of the solvent to form solid fibers that are deposited on the collector. Unlike ES, SBS does not involve the safety problem of high-voltage electricity and complex electric field designs. It also offers several advantages, including higher efficiency, suitability for a broader range of solutions, and better control over the fiber structure and diameter. However, the quality and spinning efficiency of fiber fabrics are limited by the volatility of the solvent. Low-volatile solvents can make it difficult for fibers to form as the solvent does not evaporate quickly enough during spinning [23,24,25,26]. To accelerate solvent evaporation, three strategies can be employed: increasing the liquid temperature, expanding the liquid’s surface area, and speeding up the airflow across the surface of the liquid. During SBS, expanding the surface area of the liquid and increasing the airflow rate across the surface of the liquid can be accomplished by enhancing the velocity of airflow. However, an overly strong airflow produces a broken solution jet, which results in poor fiber continuity. Increasing the temperature effectively accelerates solvent evaporation by raising the molecular energy of the liquid. To address the challenge of using water as a solvent, Medeiros, the founder of SBS, suggested using a flame to heat the spinning air. This method successfully solved the issue of spinning polyvinyl alcohol with water as a solvent [27]. However, this method causes the solution to solidify prematurely, clogging the needle and disrupting continuous spinning. The use of open flames also presents serious safety risks, limiting its industrial application. Therefore, a safer and more reliable method is needed to accelerate solvent evaporation during SBS to improve production efficiency and meet industrial needs.

In this study, we developed a double-pronged solution blow spinning (DP-SBS) system. The solution jet is ejected from the nozzle by a room-temperature airflow and encounters a second airflow with a higher speed and temperature during the movement, and then the solution jet moves along the direction of the second airflow. The use of high-speed hot airflow significantly enhances solvent evaporation during spinning, while the double-pronged design prevents common needle-clogging issues caused by the heat. DP-SBS demonstrated a high production rate of water-soluble polymer nanofibers (single needle: 5.94 g/h), which is unattainable with traditional ES and SBS. Additionally, this method proves highly efficient for producing non-water-soluble polymer nanofibers, with a production rate of 7.91 g/h (single needle), the highest reported to date. Moreover, this method not only produces conventional 2D fiber membranes but also can directly prepare 3D fiber sponges. For the first time, polyethylene oxide–carboxymethyl chitosan (PEO-CS) nanofiber sponges were successfully prepared, demonstrating excellent hemostatic properties. This method can also be extended to the preparation of other 3D nanomaterials, such as mullite nanofiber sponges, which exhibit excellent high-temperature insulation properties.

## 2. Experimental Section

### 2.1. Materials

Polyvinyl alcohol (PVA, 1788), polyethylene oxide (PEO, Mw = 5,000,000), sodium alginate (SA, AR), polyacrylonitrile (PAN, Mw = 149,000~151,000), polyvinylidene fluoride (PVDF, Mw = 455,000), polytetrafluoroethylene dispersion (PTFE, 60 wt%), aluminum isopropoxide (C_9_H_21_AlO_3_, 99.99% metals basis), polystyrene (PS, General Purpose Type I), zirconium diperchlorate oxide octahydrate (ZrOCl_2_·8H_2_O, AR, 99%), aluminum nitrate nonahydrate (Al(NO_3_)_3_·9(H_2_O), AR 99%), polyvinyl pyrrolidone (PVP, Mw = 1,300,000), and tetrabutyl titanate [Ti(OBu)_4_, 99%]—all purchased from Aladdin(Shanghai Aladdin Biochemical Technology Co., Ltd., Shanghai, China). Polyethylene oxide (PEO, Mw = 600,000), polyvinyl butyral (PVB, Mw = 90,000–120,000), hyaluronic acid (HA, 97%), and calcium chloride (CaCl_2_, AR)—all purchased from Macklin (Shanghai Macklin Biochemical Technology Co., Ltd., Shanghai, China). Sodium hydroxide (NaOH, AR), phosphoric acid (H_3_PO_4_, AR), hydrochloric acid (HCl, AR), acetone (AC, AR), tetraethyl orthosilicate [(C_2_H_5_O)_4_Si, AR], ethyl alcohol (EtOH, AR), N,N-Dimethylformamide (DMF, AR)—all purchased from Sinopharm Chemical Reagent Co., Ltd. (Shanghai, China). Carboxymethyl chitosan (CS, deacetylation degree ≥ 90%) was purchased from Cool Chemistry (Kool Chemical Technology Co., Ltd., Beijing, China). Sodium citrate anticoagulant rabbit blood was purchased from Nanjing Senbeijia Biological Co., Ltd., (Nanjing, China). Thermoplastic polyurethanes (TPU) were purchased from BASF(BASF SE, Ludwigshafen, GER), Germany. Polyamide acid (PAA, 80%) was purchased from Dongguan Xinmiao New Materials Co., Ltd., (Dongguan, China). The heat gun was purchased from Fuzhou SDANLI Welding Technology Co., Ltd. (Fuzhou, China).

### 2.2. Characterization Methods

The fibers were characterized using a Phenom Pro G6 Desktop SEM (Phenom China, Shanghai, China). Measurement of 100 fibers using the Nano Measurer to obtain the average fiber diameter. The spinning process was observed using a high-speed camera (FASTCAM Mini UX100, Photron, Ltd., Tokyo, Japan) equipped with an F-bayonet lens (Noct-NIKKOR 58 mm f/1.2, Nikon Corporation, Tokyo, Japan). A contact angle (CA) test was performed on the sample using a CA testing instrument (Theta, Biolin, Sweden). FTIR spectra were measured using a Fourier-transform infrared spectrometer (IS50, Thermo Fisher Technologies Ltd., Shanghai, China) with a spectral range of 7800–350 cm^−1^. Mapping images were obtained using a scanning electron microscope (Sigma 300, Carl Zeiss AG, Oberkochen, Germany).

### 2.3. Computational Fluid Dynamics

Computational fluid dynamics simulation was carried out using the Fluent module in the commercial software ANSYS 2021 (Swanson Analysis Systems, Inc., Canonsburg, PA, USA). The turbulence model used was k-ε, the wall function was the standard wall function, and the component model used was component transfer. The initial velocity of the airflow was set to 50 m/s. An injection source of liquid-phase water (30 μm in diameter and 293 K in temperature) was added 4 cm away from the gas injection port. The model dimensions and meshing are shown in Figure S1.

### 2.4. Fiber Collector Devices

Two types of fiber collection devices were used in this study: an exhaust fan (Xingcheng Electric, Chengdu, China) and a cage (made by oneself). The exhaust fan was used to obtain two-dimensional fibrous films, such as those made from PVA, PEO, and PTFE. In contrast, the cage was employed to collect three-dimensional sponges, such as PEO-CS, PI, and mullite.

## 3. Results and Discussion

### 3.1. The Working Mechanism of DP-SBS

**Figure 1:**
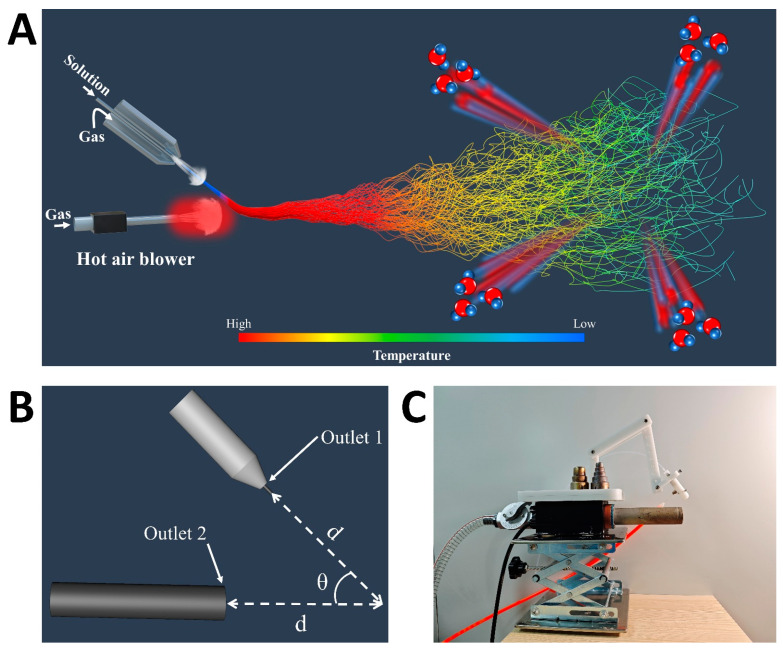
Design of DP-SBS: (**A**) schematic illustration of DP-SBS equipment; (**B**) schematic illustration of angle and distance between two nozzles; (**C**) optical image of DP-SBS equipment.

Figure 1 presents a schematic of the DP-SBS system, consisting of two main sections. The upper section includes the solution blow spinning support framework and coaxial needle assembly. In Figure 1C, the transparent tubes represent the solution input pipe, while the orange tubes indicate the airflow input pipe. The lower section houses the heat gun and its supporting structure, with the primary function of the heat gun being to maintain optimal temperature during the spinning process. The system height is adjustable via the support structure, allowing for the precise control and optimization of spinning parameters. Initially, the precursor solution is ejected under the drive of a room-temperature airflow (first airflow) to form a solution jet, which then merges with the high-speed hot airflow (second airflow). Since the velocity of the second airflow is faster than the first, the solution jet is deflected and moves with the second airflow. During the spinning process, the solution jet was generated under the action of room-temperature airflow, preventing premature solution solidification and unstable spinning due to heating (Figure S2). Additionally, the second airflow introduces higher temperatures during the propagation phase, accelerating solvent evaporation. This enables DP-SBS to address the challenge of spinning polymer solutions with low solvent volatility, producing high-quality polymer nanofiber fabrics. Most importantly, this method accommodates higher solution feeding speeds while producing higher-quality nanofiber fabrics, enabling a higher production rate.

**Figure 2:**
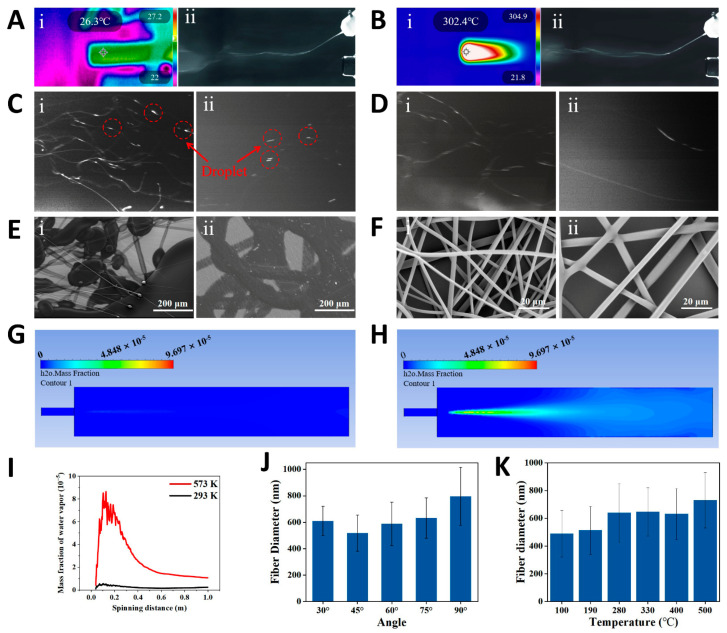
Blow spinning of water-soluble polymer (PVA and PEO) solutions. Blow spinning process using (**A**) room-temperature airflow and (**B**) hot airflow—(**i**) infrared image and (**ii**) high-speed camera image of PVA solution jets. High-speed camera images (near the collector) of spinning with (**C**) room-temperature airflow and (**D**) hot airflow—(**i**) PVA, (**ii**) PEO. (**E**) SEM images of fibers obtained by (**E**) room-temperature airflow and (**F**) hot airflow—(**i**) PVA, (**ii**) PEO. CFD simulations of the water vapor distribution under (**G**) room-temperature airflow (293 K) and (**H**) hot airflow (593 K) at the same airflow rate (water particles as liquid-phase injection source). (**I**) Mass fraction of water vapor distribution along the spinning axis as a function of spinning distance. Influence of (**J**) the angle between two airflows and (**K**) temperature of hot airflow on the diameter of PVA nanofibers.

We captured high-speed images of the spinning of two water-soluble polymer solutions, polyvinyl alcohol (PVA) and PEO, at both the outlet and the receiving end under heated and unheated conditions. High-speed images of the initial phase show significant deflection of the solution jet at the intersection of the two airflows, moving toward the hot airflow (Figure 2A,B). This indicates that the second airflow has a higher velocity than the first and becomes dominant after convergence. High-speed images of the spinning near the collector are shown in Figure 2C,D. The images clearly show visible liquid droplets during room-temperature spinning, whereas in the hot-air spinning process, almost no droplets are observed. To further investigate the effect of hot airflow on fiber morphology, we used scanning electron microscopy (SEM) to observe the micromorphology of water-soluble polymer fibers. The SEM images show large areas of polymer droplets and partially solidified fibers (Figure 2E), indicating that room-temperature spinning does not produce satisfactory fiber structures. In contrast, hot-air spinning produces a uniform, smooth, and droplet-free fiber structure (Figure 2F). The influence of hot airflow on solvent evaporation was further analyzed using computational fluid dynamics (CFD) simulation (Figure S3). The simulation results reveal a significant difference in water vapor distribution between room-temperature airflow (293 K) and hot airflow (573 K) (Figure 2G,H). By integrating the water vapor distribution curve, the total mass fraction of water vapor within 1 m (axial direction of spinning) is calculated as 2.14 × 10^−6^ (293 K) and 2.7 × 10^−5^ (573 K), indicating that the evaporation rate at 573 K is an order of magnitude higher than at room temperature. For SBS, hot airflow accelerates solvent evaporation, resulting in higher production rates and improved spinnability for solutions like water-soluble polymers with a slower solvent evaporation rate.

In order to study the effect of temperature on liquid evaporation, the governing equation of liquid evaporation is briefly described [28]. The mass transfer rate is as follows:

(1) $$J=\beta \sqrt{\frac{M}{2\pi RT_{\mathrm{sat}}}}L_{a}(\frac{c_{v}\rho_{l}}{\rho_{l}-C_{v}})\frac{T-T_{\mathrm{sat}}}{T_{\mathrm{sat}}}$$

where *β* is close to 1.0, near the equilibrium condition; *M* is the relative molecular mass; *L_a_* is the latent heat of evaporation; *ρ_l_* is the liquid density; *R* is the gas constant; and saturation temperature (*T_sat_*) corresponds to the partial pressure of water vapor (*p_v_*). This is calculated by the following formula:

(2) $$T_{\mathrm{sat}}=b_{1}+b_{2}\ln p_{v}+b_{3}{\left(\ln p_{v}\right)}^{2}$$

These include *b_1_* = 35.96, *b_2_*= −1.87, and *b_3_*= 1.17. Assuming that water vapor is an ideal gas, the partial pressure of water vapor (*p_v_*) and concentration (*C_v_*) converted by the Clapeyron equation are as follows:

(3) $$p_{v}=C_{v}R$$

From Equations (1)–(3), it can be concluded that the driving force for mass transfer is the difference between the partial pressure of saturated water vapor at the liquid–gas interface and the water vapor partial pressure in the air near the interface. This difference is directly related to the droplet evaporation rate, and the partial pressure of saturated water vapor is strongly influenced by temperature. As the temperature rises, the partial pressure of saturated water vapor increases due to the higher kinetic energy of the molecules, which enhances the likelihood of water vapor entering the gas phase, thus raising its pressure in the gas phase. This principle allows us to further verify the effect of temperature on the solution blow during volatilization. As temperature increases, the partial pressure of saturated water vapor rises, increasing the difference between it and the water vapor partial pressure in the surrounding air, which drives mass transfer. This means that higher temperatures accelerate the mass transfer process, leading to a faster rate of liquid evaporation. Next, we considered the effect of different spinning parameters on fiber morphology. First, we studied the effect of the angle on the fiber diameter. Results show that when the angle between the two airflows is 45°, the average fiber diameter is thinnest (Figure 2J). Second, we varied the temperature of the second airflow and examined the changes in average fiber diameter at different temperatures. The results show that as temperature increases, the average fiber diameter also increases. This is due to accelerated solvent evaporation and premature solidification of the solution jets, resulting in thicker fibers (Figure 2K). Additionally, we studied the effects of varying second airflow velocity and solution feeding speed on the average diameter of PVA fibers. The results indicate that higher second airflow velocity results in a thinner average fiber. Similarly, a faster solution injection rate results in thicker fibers (Figure S4). These studies provide valuable insights into fiber diameter regulation and offer important guidance for optimizing the spinning process.

### 3.2. Universality, Reasonable Temperature, and Productivity of DP-SBS

**Figure 3:**
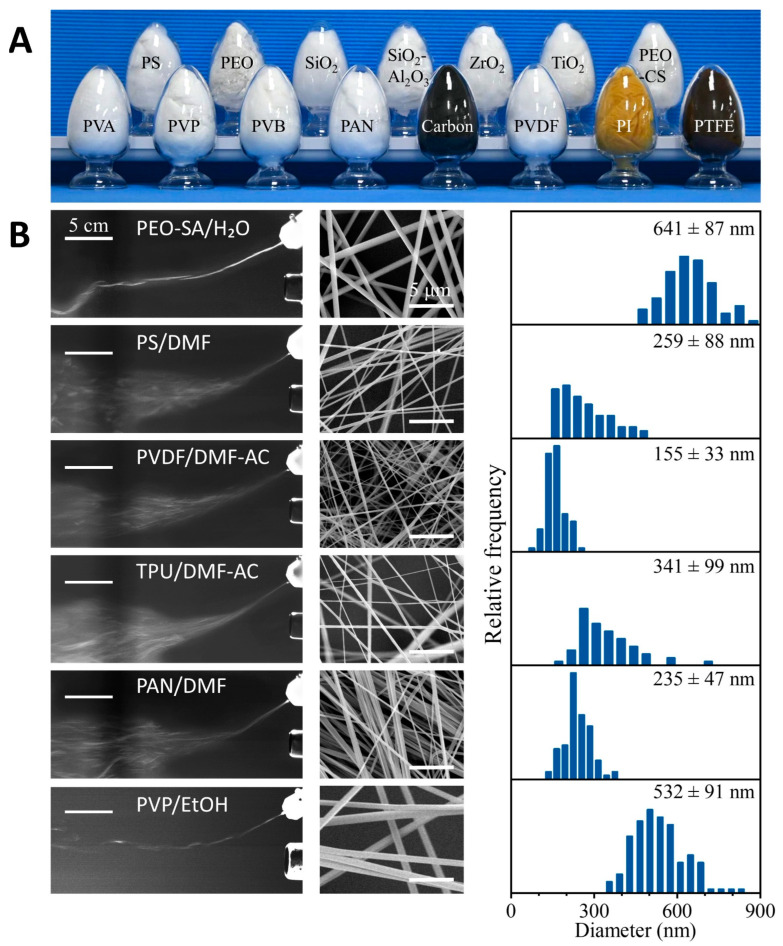
Universality of DP-SBS: (**A**) optical image of various nanofiber materials manufactured using DP-SBS; (**B**) liquid jets of various polymer solutions captured by high-speed cameras (**left column**), SEM images (**middle column**), and diameter distribution (**right column**) of polymer fibers.

To verify that DP-SBS can produce various nanofibers, we used it to prepare a range of polymer nanofibers—including PVA, polyvinyl pyrrolidone (PVP), polyvinyl butyraldehyde (PVB), polyimide (PI), polyvinylidene fluoride (PVDF), PEO, polystyrene (PS), and polyacrylamide (PAN)—as well as carbon, ceramic, and nanofiber composites (Figure 3A). These polymer solutions, using different solvents, demonstrated high stability during spinning (e.g., PEO-SA/H_2_O, PS/DMF, PVDF/DMF-AC, TPU/DMF-AC, PAN/DMF, and PVP/EtOH). All these solutions were extruded through a needle, formed into solution jets under the shear force of the airflow, and then intersected with the heated second airflow. As the solution jets move with the second airflow, the solvent evaporates continuously to form uniform nanofibers (Figure 3B, left column). The resulting nanofibers exhibit uniform morphology (Figure 3B, right column). By heat-treating certain nanofiber precursors, carbon nanofibers, PI nanofibers, polytetrafluoroethylene (PTFE) nanofibers, and ceramic nanofibers can also be obtained. These results demonstrate that the process is compatible with most spinning solvent systems and can prepare various nanofibers with different properties. Therefore, as a mature spinning technology, DP-SBS offers broad application potential.

**Figure 4:**
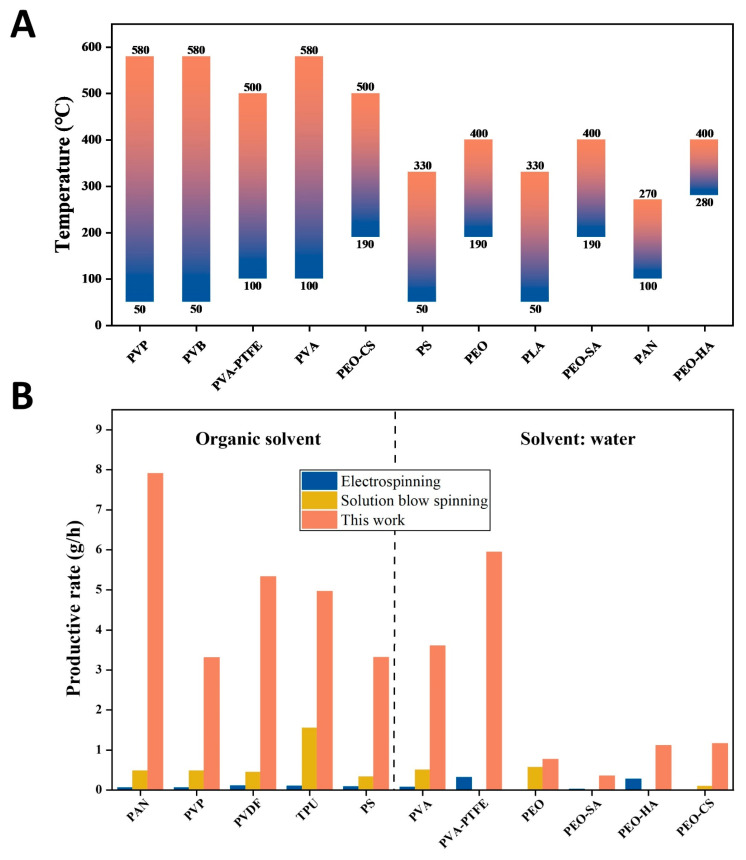
The reasonable temperature and productivity of DP-SBS: (**A**) reasonable spinning temperatures for different polymers; (**B**) comparison of production rates (single needle) between DP-SBS, ES, and SBS for different polymers.

The temperature of the airflow is a very important parameter for DP-SBS so we studied the effect of this parameter on fiber forming. Using PAN as an example, when the temperature of the airflow was set to 48 °C, we observed that a large number of droplets—due to incomplete solvent evaporation—were formed on the fiber surface. At temperatures between 100 °C and 270 °C, uniform, droplet-free nanofibers can be obtained. If the temperature is further increased to 400 °C, the fiber curls and its diameter becomes significantly thicker (Figure S5), which is mainly due to the excessively high temperature that intensifies the phase separation of the solution jets. This demonstrates that in DP-SBS spinning, the temperature must be controlled within a reasonable range. Moreover, different polymers require specific temperature ranges. To determine the optimal temperature for each polymer solution, we conducted a series of spinning experiments at various temperatures and identified the appropriate temperature for each polymer (Figure 4A). These results provide a valuable reference for optimizing the production of various polymer nanofibers.

For DP-SBS, increasing the temperature is beneficial to improving the production rate. Our research showed that at the same solution feeding speed, the morphology of fibers obtained by blow spinning with room-temperature airflow and hot airflow is significantly different (Figure S6). Using PAN as an example, when the solution feeding speed is 54 mL/h, the blow spinning with hot airflow can obtain fibers with a smooth surface and no droplets. However, there are a large number of droplets in the fiber fabrics obtained by blow spinning with room-temperature airflow at the same solution feeding speed. Figure 4B shows a comparison of production rates (single needle) between DP-SBS, ES, and SBS (Table S1) [29,30,31,32,33,34,35,36,37,38,39,40,41,42,43,44,45,46]. The maximum production rate of PAN fibers using DP-SBS reached 7.91 g/h, an order of magnitude higher than traditional ES and SBS (single needle). This clearly demonstrates that DP-SBS has the potential for large-scale nanofiber production. Additionally, this technology not only increases fiber yield but also enhances the quality of the fibers, making it highly promising for industrial applications.

### 3.3. Application Demonstration of Several Nanofiber Materials Prepared by DP-SBS

**Figure 5:**
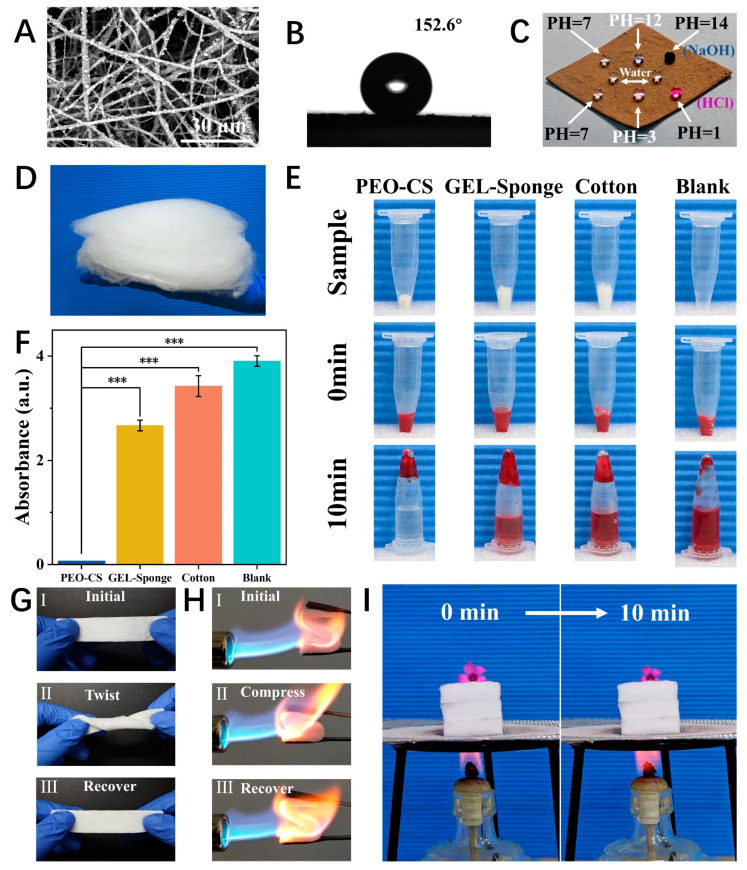
Applications of DP-SBS: (**A**) SEM image and (**B**) water contact angle (CA) of PTFE fiber membrane; (**C**) optical image of hydrophobicity of PTFE fiber membranes for solutions with different pH levels; (**D**) optical image of PEO-CS nanofiber sponges; (**E**) optical images of in vitro blood clotting experiments, (n = 3), *** *p* < 0.001; (**F**) absorbance of the supernatant of blood after 10 min; (**G**) flexibility demonstration of mullite sponge; (**H**) flexibility demonstration of mullite sponge in a butane flame; (**I**) thermal insulation demonstration of mullite sponge.

The nanofibers prepared by DP-SBS exhibit good quality. For instance, the precursor of the PTFE nanofiber membrane (PVA/PTFE) shows uniform fibers (Figure S7). After heat treatment, the fiber surface became rough (Figure 5A) due to the decomposition of PVA and exhibited superhydrophobic properties (CA = 152.6°) (Figure 5B). Additionally, the membrane is lipophilic, allowing oil to instantly penetrate when dropped onto the surface (Figure S8C). Its hydrophobic and lipophilic properties are better than those of commercial PTFE membranes (Figure S8A,B). These properties are essential for wastewater purification and oil–water separation as the wetting behavior allows the oil droplets to easily penetrate the membrane while blocking water. Moreover, PTFE nanofiber membranes remain highly hydrophobic even in strong acids and alkalis (Figure 5C), indicating their high chemical stability and potential for superhydrophobic materials.

Complex turbulence entangles the nanofibers with each other, which is conducive to the formation of 3D nanofiber aerogels. Therefore, DP-SBS has a unique advantage in preparing 3D nanofiber materials. Three-dimensional nanofiber materials possess advantages over two-dimensional ones, such as a highly porous fiber network and a large specific surface area, attracting significant attention. We prepared PEO-CS nanofiber sponges using DP-SBS and studied their hemostatic properties. CS is a natural polymer with excellent hemostatic and antibacterial effects, making it an ideal wound dressing. Here, we made CS aqueous solution spinnable by adding a small amount of PEO (PEO:CS = 1:13), based on which the PEO-CS nanofiber sponge was prepared (Figure 5D). The chemical composition of the sponge was investigated by FTIR spectroscopy (Figure S9). PEO-CS sponges exhibited excellent blood clotting properties (Figure 5E,F), outperforming traditional hemostatic materials like gelatin sponge and hemostatic cotton, with a shorter blood clotting time of 258 ± 21 s (Figure S10). Additionally, the hemolysis rate of PEO-CS sponges is 3.33% (Figure S11), below the 5% international safety standard, making it a safe and effective hemostatic material.

We also prepared mullite ceramic nanofiber sponges using the DP-SBS. Elemental analysis confirmed the material is composed of O, Si, and Al, with the ratio of Al and Si being 3:1 (Figure S12). The flexibility of the sponge was verified by twisting it 360° in situ (Figure 5G). The compression and recovery tests under a butane flame demonstrated that the ceramic sponges have excellent high-temperature resistance and thermomechanical properties (Figure 5H). Additionally, a flower was placed on a 3-centimeter-thick mullite sponge and heated with an alcohol lamp. After 10 min, the flower changed only slightly, proving the excellent insulating properties of the ceramic aerogel (Figure 5I). In summary, nanofiber materials prepared using DP-SBS exhibit excellent performance and broad application prospects in fields like oil–water separation, hemostasis, and heat insulation.

This technology not only achieves the combination of high quality and high production rates for nanofibers but also provides a reliable strategy for large-scale production. With further research and optimization, DP-SBS is expected to bring innovation and change to more fields, providing more efficient and multifunctional nanofiber materials for future industrial production and daily life.

## 4. Conclusions

In summary, we developed a DP-SBS system. This spinning system significantly accelerates solvent evaporation, and its double-pronged design effectively prevents needle clogging caused by heating. DP-SBS enables a high production rate of water-soluble polymer nanofibers, achieving a production rate of up to 5.94 g/h, far surpassing the capabilities of traditional ES and SBS methods. This technology is also highly efficient for producing non-water-soluble polymer nanofibers, with a production rate of up to 7.91 g/h, the highest value reported to date. In addition, this approach can be used not only to fabricate common 2D fiber membranes but also to produce 3D fiber sponges in a single step. For the first time, the preparation of PEO-CS nanofiber sponges was achieved, demonstrating excellent hemostatic properties in medical hemostasis. This method can also be extended to the production of other 3D nanomaterials, such as mullite sponges, which exhibit outstanding high-temperature insulation properties. This innovative spinning method will provide strong support for the global application and widespread use of nanofibers.

## Data Availability

The original contributions presented in this study are included in the article/Supplementary Materials. Further inquiries can be directed to the corresponding author(s).

## Supplementary Materials

The following supporting information can be downloaded at https://www.mdpi.com/article/10.3390/nano15080578/s1, Figure S1: CFD simulations of (**a**) Model dimensions. (**b**) Meshing. Figure S2: Solution blow spinning with hot airflow (PVA). (**A**) High-speed camera images of PVA solution jets. (**B**) SEM images of PVA fibers. Figure S3: CFD simulations of the airflow velocity distribution with (**A**) room temperature airflow (293 K) and (**B**) hot airflow (593 K). CFD simulations of the distribution of temperature with (**C**) room temperature airflow (293 K) and (**D**) hot airflow (593 K). Figure S4: Influence of different spinning parameters on PVA fiber diameter: (**A**) airflow velocity, (**B**) solution feeding speed. Figure S5: SEM images of PAN nanofibers obtained using airflow at different temperatures. Figure S6: The SEM images of the fibers were compared by using higher solution feeding speed under high temperature airflow(left) and room temperature airflow(right). PAN (54 mL/h), PVP (48 mL/h), PS (24 mL/h), PVDF (48 mL/h), TPU (48 mL/h). Figure S7: SEM image of PTFE nanofiber precursor. Figure S8: Contact angle (CA) of different PTFE samples for water and oil. (**A**) CA of commercial PTFE film for water. (**B**) CA of commercial PTFE film for oil. (**C**) CA of PTFE prepared in this study for oil. Figure S9: FTIR spectra of PEO, CS, and PEO-CS. Figure S10: Comparison of blood clotting time between PEO-CS and traditional hemostatic mate-rials. (**A**) Optical images of blood clotting test. (**B**) Blood clotting times of different materials (n=3). ** *p* < 0.01, *** *p* < 0.001. Figure S11: (**A**) Photographs of hemolysis. (**B**) Hemolysis ratio (n = 3). *** *p* < 0.001 Figure S12: Elemental analysis of mullite sponge. (**A**) SEM images of a single fiber of mullite sponge and mapping images of different elements. (**B**) Composition and elemental content of mullite sponge. Table S1: Comparison of the production rate of polymer nanofibers using different spinning techniques.
