# Supplementary material for: Hot-Air Spinning Technology Enables the High-Efficiency Production of Nanofiber
Source: Nanomaterials (Basel). 2025 Apr 11;15(8):578. doi: 10.3390/nano15080578 (PMC12029198; doi:10.3390/nano15080578)
Supplement: Supplementary file 1 [file nanomaterials-15-00578-s001.zip › nanomaterials-3541451-supplementary.pdf]

Supplementary Materials

Preparation of Nanofibers

Polyvinyl alcohol (PVA) solution: Dissolve 10 g of PVA in 90 g of deionized water, The mixed solution was magnetically stirred at 60°C for 12 hours to obtain a homogeneous solution. Polyethylene oxide (PEO) solution: Dissolve 6.5 g of PEO (Mw = 600,000) in 100 g of deionized water. The mixed solution was magnetically stirred at 40°C for 6 hours to obtain a homogeneous solution. Polyvinyl pyrrolidone (PVP) solution: Dissolve 10 g of PVP in 90 g of ethyl alcohol (EtOH). Polyvinyl butyral (PVB) solution: Dissolve 10 g of PVB in 90 g of EtOH. Polyvinylidene fluoride (PVDF) solution: Dissolve 6 g of PVDF into a mixture of 17 g of N,N-Dimethylformamide (DMF) and 17 g of acetone (AC). Polystyrene (PS) solution: Dissolve 3 g of PS in 17 g of DMF. Polyacrylonitrile (PAN) solution: Dissolve 15 g of PAN in 75 g of DMF and stir for 6 h. Polyethylene oxide-sodium alginate (PEO-SA) solution: Dissolve 0.075 g of PEO (Mw = 5,000,000) and 0.3 g of SA in 10 g of deionized water. Polyethylene oxide-carboxymethyl chitosan (PEO-CS) solution: Dissolve 0.075 g of PEO (Mw = 5,000,000) and 0.85 g of CS in 10 g of deionized water. Polyethylene oxide-hyaluronic acid (PEO-HA) solution: Dissolve 0.075 g of PEO (Mw = 5,000,000) and 0.85 g of HA in 10 g of deionized water. Thermoplastic polyurethanes (TPU) solution: Dissolve 6 g of TPU in 17 g of DMF and 17 g of AC.

Polyimide (PI) nanofibers

Mix 5 g of polyamide acid (PAA) solution with 25 g of DMF and stir for 30 minutes to homogenize. Then, add 0.2 g of PEO (Mw = 5,000,000) to the PAA/DMF solution, the mixed solution was magnetically stirred at 40°C for 5 h to obtain a homogeneous solution. Afterward, PAA nanofibers were obtained via the DP-SBS process. The nanofiber was treated at 300°C for 10 min with a heating rate of 5°C min^{-1} in the air before cooled down in the furnace

Carbon nanofibers

First, the PAN nanofibers were treated at 280 °C by setting the heating rate to 5°C min^{-1} and by using a holding time of 1 hour. Afterward, the stabilized PAN nanofibers were then heated in a nitrogen atmosphere to 1000 °C at a rate of 5 °C min^{-1} and held for 1 h.

Polytetrafluoroethylene dispersion (PTFE) nanofibers

Dissolve 1.5 g of PVA in 8.5 g of deionized water and subsequently heat the solution in a water bath to 60°C with stirring for 6 hours. Then, add 10 g of PTFE dispersion to the PVA solution and stir for 1 h. Afterward, the solution was used to prepare precursor nanofiber via the DP-SBS process. The nanofiber was treated at 390°C for 10 min with a heating rate of 3°C min^{-1} in the air before cooled down in the furnace.

Mullite sponge

First, 2.2 g of tetraethyl orthosilicate [$(C_2H_5O)_4Si$], 3.38 g of aluminum nitrate nonahydrate [$Al\ (NO_3)_3{\cdot}9(H_2O)$], and 4.2 g of aluminum nitrate nonahydrate ($C_9H_{21}AlO_3$) were subsequently added to the 10 g of water, the solution was magnetically stirred for 6 hours at room temperature. Then, add 19.78 g of 15% PVA solution and continue stirring for 1 hour. Afterward, the solution was used to prepare precursor nanofiber sponges via the

DP-SBS process. The nanofiber sponge was treated at 1100°C for 1 hours with a heating rate of 5°C min^{-1} in the air before cooled down in the furnace.

Zirconium dioxide (ZrO_2) nanofibers

Add 4 g of PVP and 8 g of zirconium diperchlorate oxide octahydrate ($ZrOCl_2·8H_2O$) to a mixture of 20 g of deionized water and 20 g of ethanol, the solution was magnetically stirred for 6 hours at room temperature. Afterward, the solution was used to prepare precursor nanofiber sponges via the DP-SBS process. The nanofiber sponge was treated at 800°C for 2 hours with a heating rate of 5°C min^{-1} in the air before cooled down in the furnace.

Titanium dioxide (TiO_2) nanofibers

Add 4 g of PVP and 16 g of Ti $(OBu)_4$ to a mixture of 20 g of deionized water and 20 g of ethanol, the solution was magnetically stirred for 6 hours at room temperature. Afterward, the solution was used to prepare precursor nanofiber sponges via the DP-SBS process. The nanofiber sponge was treated at 450°C for 2 hours with a heating rate of 5°C min^{-1} in the air before cooled down in the furnace.

Silica (SiO_2) nanofibers

Dissolve 1.52 g of PVA in 10 g of deionized water and subsequently heat the solution in a water bath to 60°C with stirring for 12 hours. Then, add 10 g of tetraethyl orthosilicate [$(C_2H_5O)_4Si$] and 0.2 g of phosphoric acid (H_3PO_4), the solution was magnetically stirred for 1 hours at room temperature. Afterward, the solution was used to prepare precursor nanofiber sponges via the DP-SBS process. The nanofiber sponge was treated at 600°C for 2 hours with a heating rate of 2°C min^{-1} in the air before cooled down in the furnace.

In Vitro Whole Blood Clotting Test

Prepare different types of samples (10mg) and put it into centrifuge tube. The whole blood (100 μL) was dropped onto each sample and calcium chloride ($CaCl_2$) solution (10 μl, 0.2 M) was immediately added. After 10 min, the deionized water (1 mL) was added to the centrifuge tube to release the uncoagulated blood components, and the centrifuge tube was observed upside down to check the clot status. Finally, the absorbance of the supernatant was measured at 540 nm (a lower OD indicates a faster clotting rate). All experiments were repeated three times.

Blood Clotting Time Test

1 mL of anticoagulated rabbit blood (with sodium citrate) was added to centrifuge tubes containing 10 mg of PEO-CS, medical gelatin sponge, or hemostatic cotton, respectively. A blank centrifuge tube was used as the control, and 30 μL of 0.25M CaCl2 solution was then added. The tube was inverted every 15 seconds, and the blood clotting time was recorded each time (n = 3).

Hemolytic Test

Red blood cells were obtained from rabbit whole blood by centrifugation (3000 rpm, 10 min) and washed with saline and diluted to 5% (v/v). 20 mg of each sample was added to 2 mL of red blood cell diluent and incubated at 37 °C for 24 h. 200 μL of Triton x-100 (0.1%) and saline were added to 2 mL of red blood cell diluent as negative and positive controls, respectively. Then all the treated red blood cell diluents were centrifuged (3000 rpm, 10 min), and the absorbance of supernatant at 540 nm was measured. The hemolysis ratio was calculated as follows:

$$hemolysis\ ratio(\%) = \frac{Tx - Tn}{Tt - Tn} \times 100\% \quad (1)$$

(T_x, T_n, T_t are the absorbance of supernatant treated with different samples, normal saline, and Triton X-100 respectively).

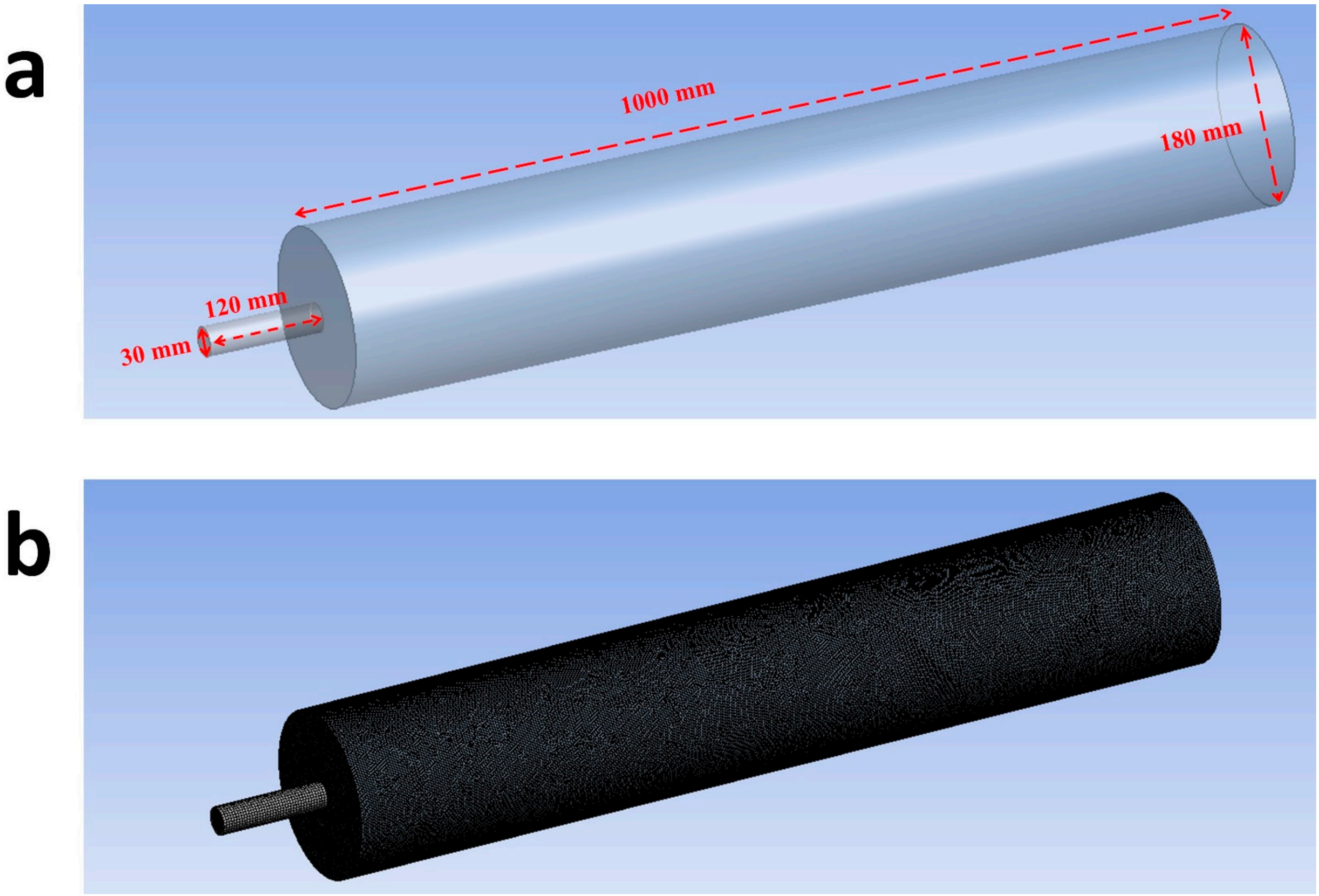

Figure S1. CFD simulations of (A) Model dimensions. (B) Meshing.

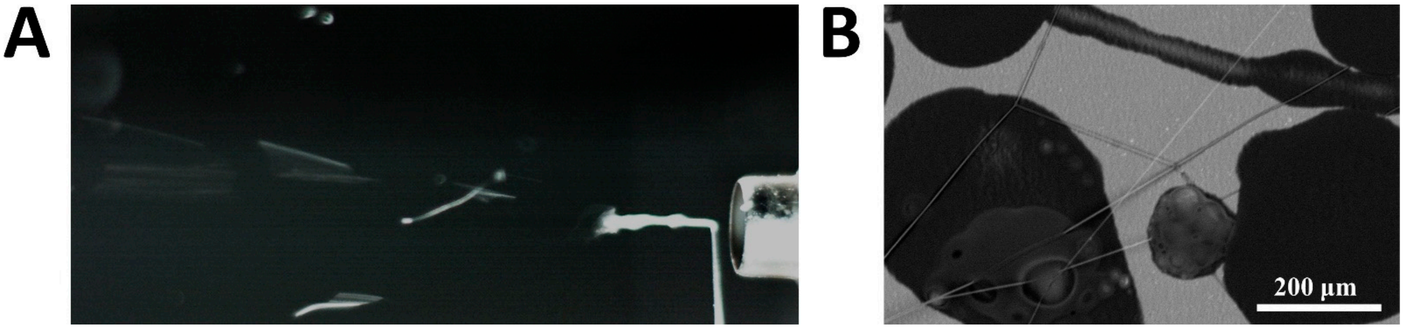

Figure S2. Solution blow spinning with hot airflow (PVA). (A) High-speed camera images of PVA solution jets. (B) SEM images of PVA fibers.

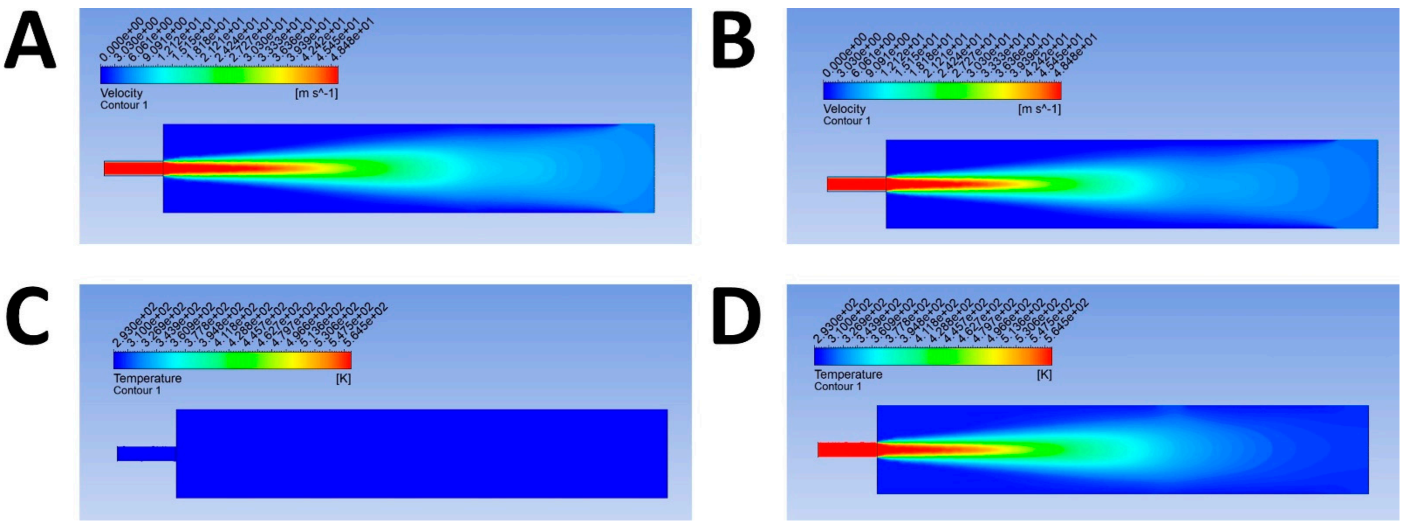

Figure S3. CFD simulations of the airflow velocity distribution with (A) room temperature airflow (293 K) and (B) hot airflow (593 K). CFD simulations of the distribution of temperature with (C) room temperature airflow (293 K) and (D) hot airflow (593 K).

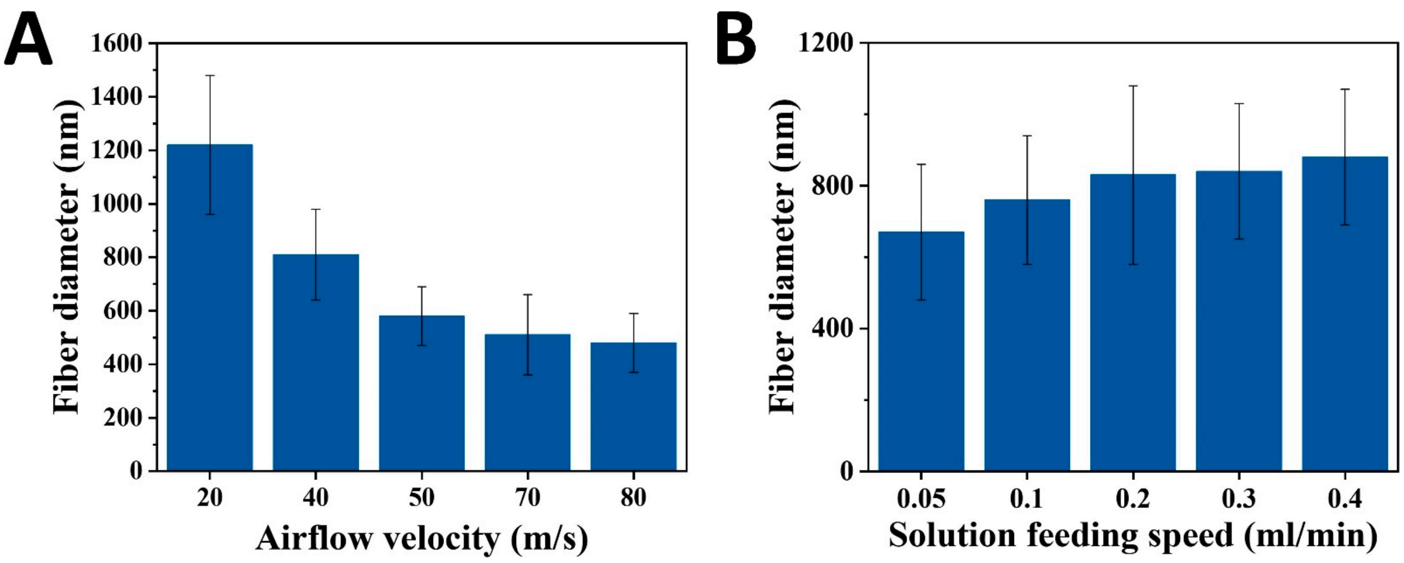

Figure S4. Influence of different spinning parameters on PVA fiber diameter: (A) airflow velocity, (B) solution feeding speed.

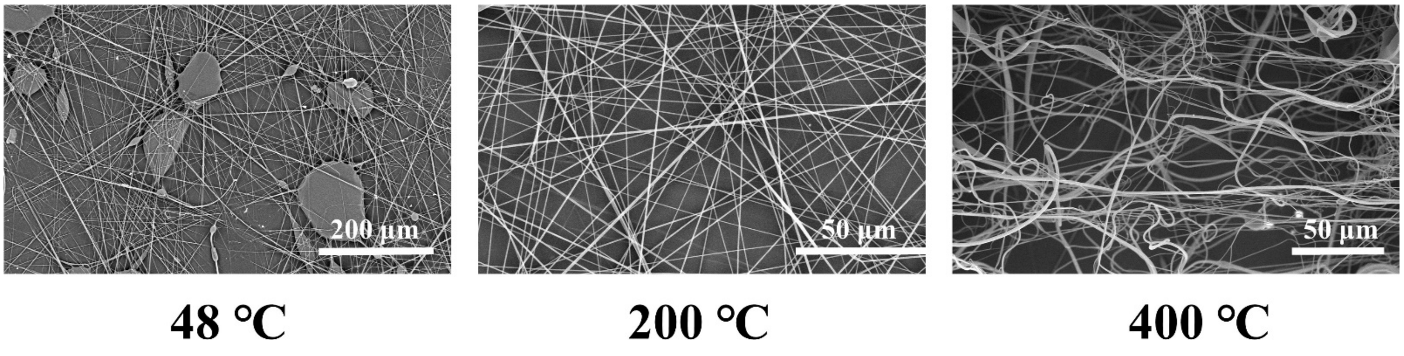

Figure S5. SEM images of PAN nanofibers obtained using airflow at different temperatures.

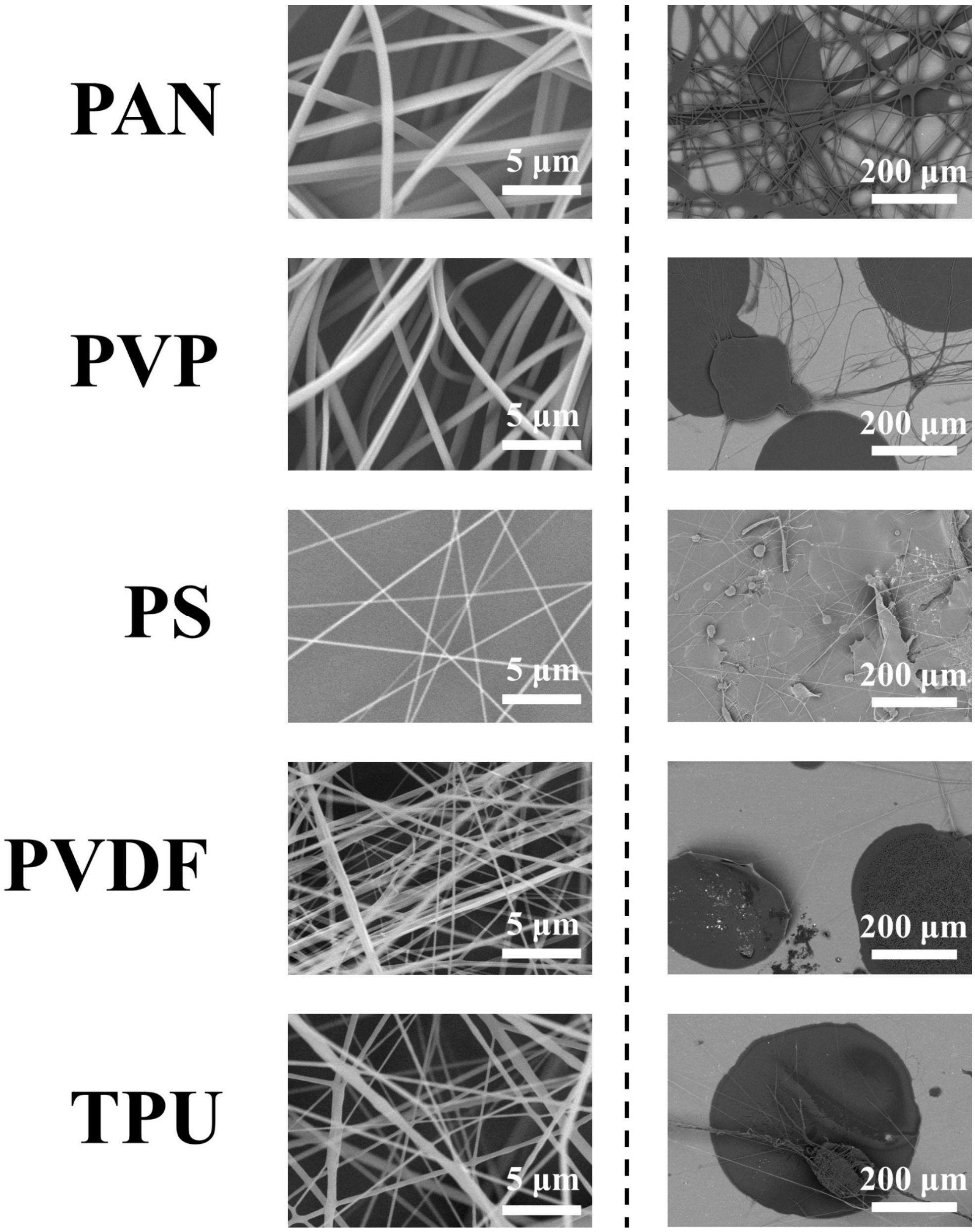

Figure S6. The SEM images of the fibers were compared by using higher solution feeding speed under high temperature airflow(left) and room temperature airflow(right). PAN (54 mL/h), PVP (48 mL/h), PS (24 mL/h), PVDF (48 mL/h), TPU (48 mL/h).

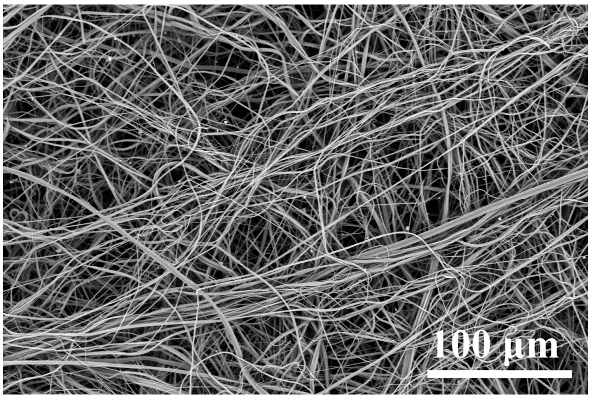

Figure S7. SEM image of PTFE nanofiber precursor.

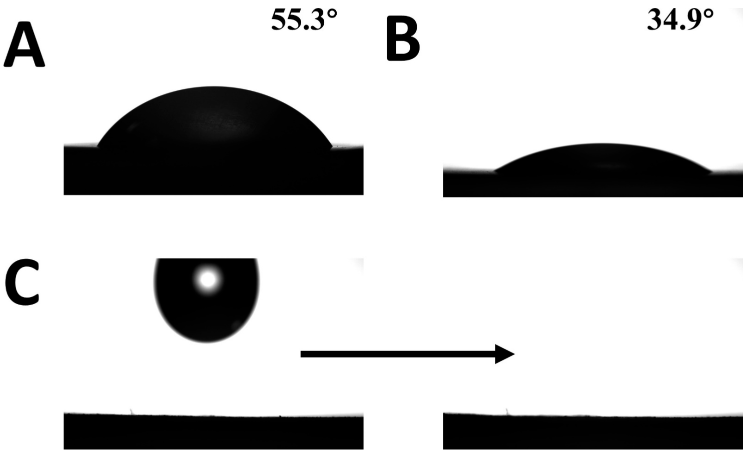

Figure S8. Contact angle (CA) of different PTFE samples for water and oil. (A) CA of commercial PTFE film for water. (B) CA of commercial PTFE film for oil. (C) CA of PTFE prepared in this study for oil.

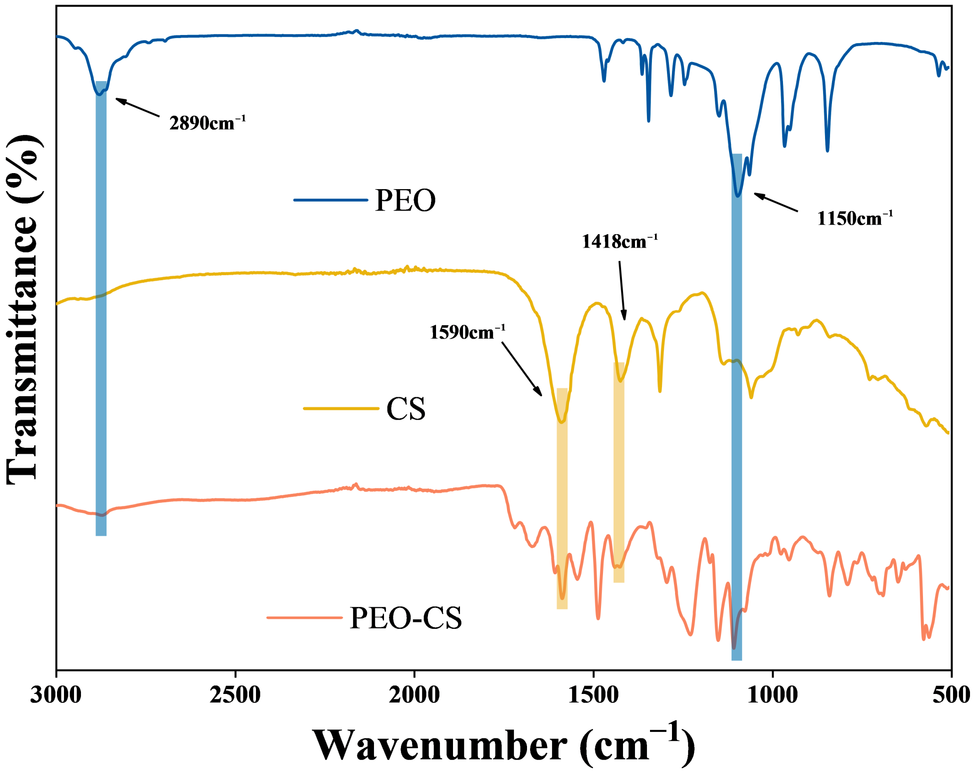

Figure S9. FTIR spectra of PEO, CS, and PEO-CS.

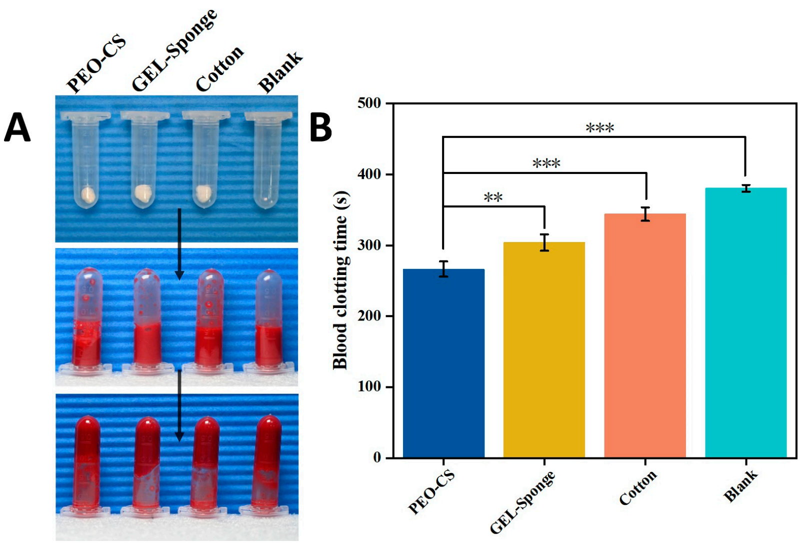

Figure S10. Comparison of blood clotting time between PEO-CS and traditional hemostatic materials. (A) Optical images of blood clotting test. (B) Blood clotting times of different materials (n=3). ***p* <0.01, ****p* <0.001.

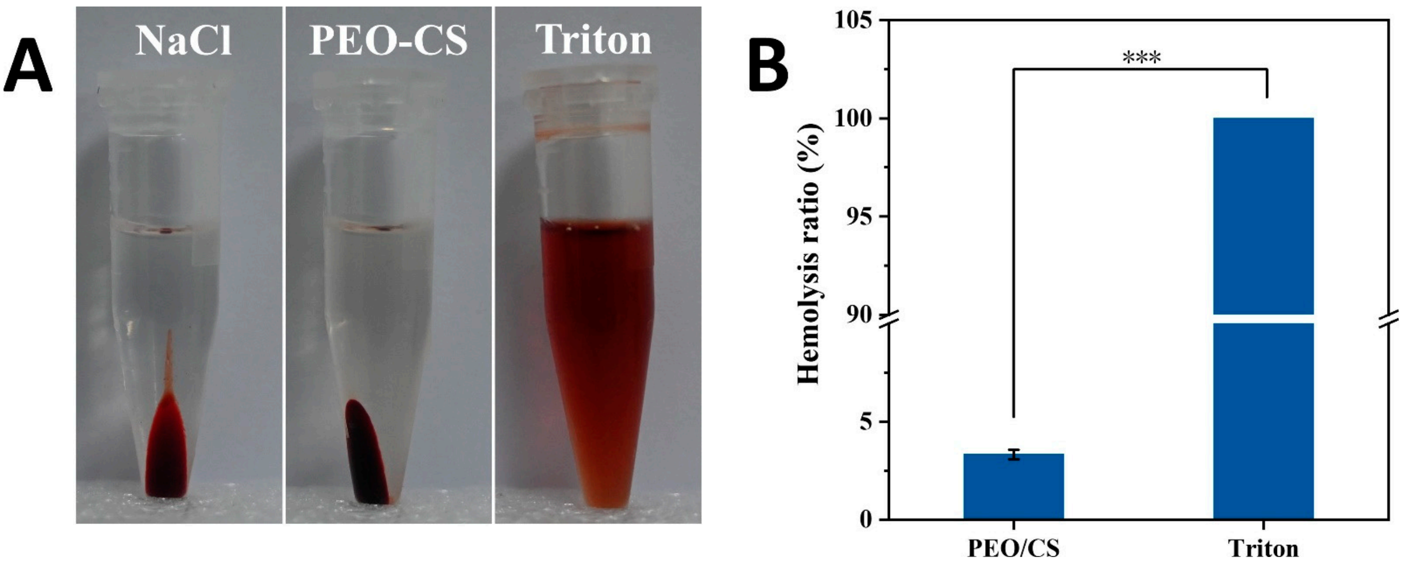

Figure S11. (A) Photographs of hemolysis. (B) Hemolysis ratio (n=3). ****p* <0.001.

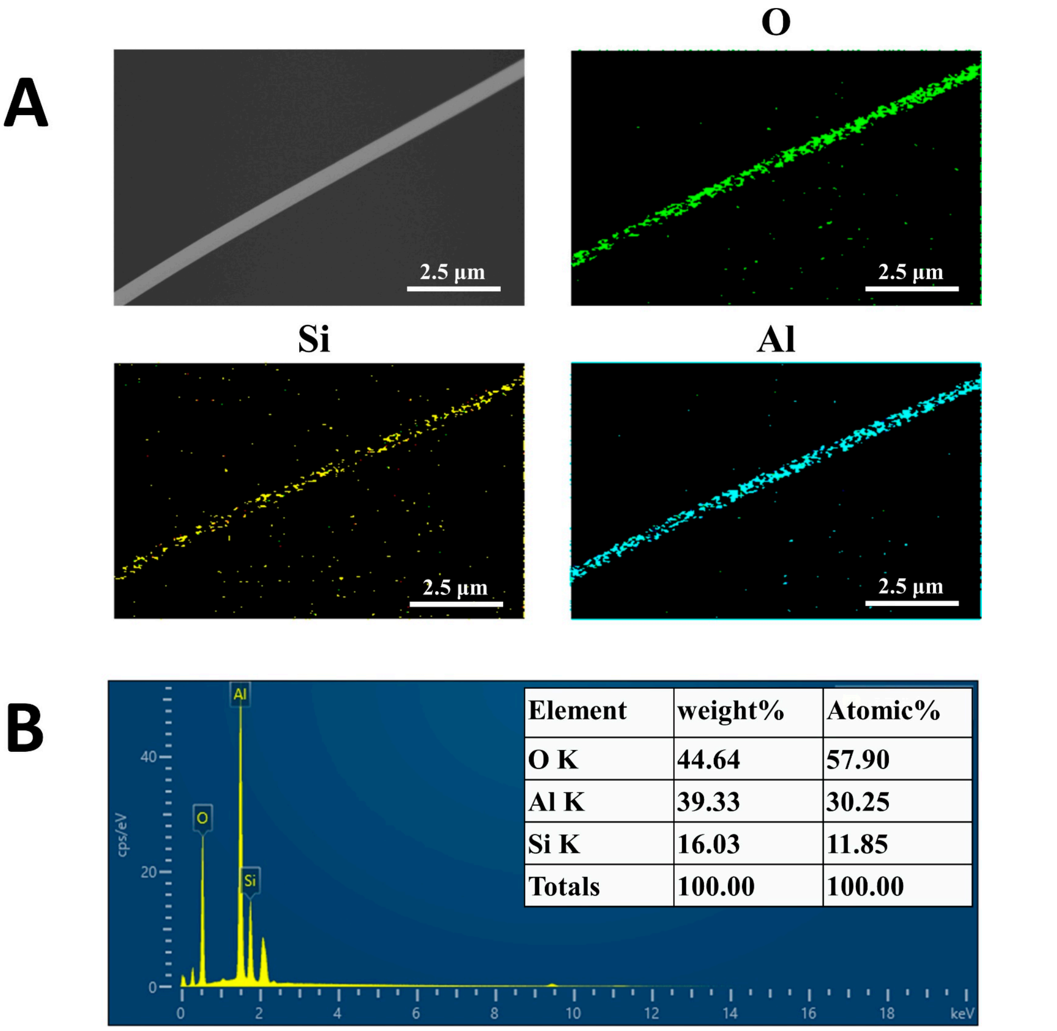

Figure S12. Elemental analysis of mullite sponge. (A) SEM images of a single fiber of mullite sponge and mapping images of different elements. (B) Composition and elemental content of mullite sponge.

Table S1. Comparison of the production rate of polymer nanofibers using different spinning techniques.

Polymer name	The production rate of ES with single-needle (g/h)	The production rate of SBS with single-needle (g/h)	The production rate of DP-SBS with single-needle (g/h)
PAN	0.058(ref.[1])	0.48(ref.[2])	7.91
PVP	0.058(ref.[3])	0.483(ref.[4])	3.312
PVDF	0.111(ref.[5])	0.444(ref.[6])	5.328
TPU	0.1035(ref.[7])	1.5525(ref.[8])	4.968
PS	0.0829(ref.[9])	0.3318(ref.[10])	3.31848
PVA	0.075(ref.[11])	0.72(ref.[12])	3.6
PVA-PTFE	0.99(ref.[13])		5.94
PEO	0.0095(ref.[14])	0.5702(ref.[14])	0.7722
PEO-SA	0.0195(ref.[15])		0.351
PEO-HA	0.2775(ref.[16])		1.11
PEO-CS	0.0000485(ref.[17])	0.097(ref.[18])	1.1655

References

1. Dong, S.X.; Li, J.; Zhang, S.; Li, N.; Li, B.; Zhang, Q.L.; Ge, L.Q. Excellent microwave absorption of lightweight PAN-based carbon nanofibers prepared by electrospinning. *Colloid Surf. A-Physicochem. Eng. Asp.* **2022**, *651*, 8.
2. Ergün, A.B.; Sevim, A.M.; Kiliç, A.; Gül, A. Metallophthalocyanine/polyacrylonitrile nanofibers by solution blow spinning technique for enhanced photocatalytic activity by visible light. *J. Appl. Polym. Sci.* **2021**, *138*, 9.
3. Elishav, O.; Beilin, V.; Rozent, O.; Shter, G.E.; Grader, G.S. Thermal shrinkage of electrospun PVP nanofibers. *J. Polym. Sci. Pt. B-Polym. Phys.* **2018**, *56*, 248-254.
4. Freire, L.A.; Lemos, A.C.C.; Miranda, K.W.E.; da Silva, J.P.; de Oliveira, J.E. Statistical optimization for preparing nanofibrous mats of polybutylene adipate co-terephthalate/poly(vinylpyrrolidone) blends by solution blow spinning. *Polym. Eng. Sci.* **2022**, *62*, 2511-2523.
5. Nie, Y.L.; Zhang, S.H.; He, Y.; Zhang, L.Y.; Wang, Y.Q.; Li, S.S.; Wang, N. One-step modification of electrospun PVDF nanofiber membranes for effective separation of oil-water emulsion. *New J. Chem.* **2022**, *46*, 4734-4745.
6. Liu, R.Q.; Wang, X.X.; Fu, J.; Zhang, Q.Q.; Song, W.Z.; Xu, Y.; Chen, Y.Q.; Ramakrishna, S.; Long, Y.Z. Preparation of Nanofibrous PVDF Membrane by Solution Blow Spinning for Mechanical Energy Harvesting. *Nanomaterials* **2019**, *9*, 11.
7. Li, B.Y.; Liu, Y.H.; Wei, S.; Huang, Y.T.; Yang, S.W.; Xue, Y.; Xuan, H.Y.; Yuan, H.H. A Solvent System Involved Fabricating Electrospun Polyurethane Nanofibers for Biomedical Applications. *Polymers* **2020**, *12*, 12.
8. Ramos, P.; Calvo-Correas, T.; Eceiza, A.; González-Benito, J. Nonwoven Mats Based on Segmented Biopolyurethanes Filled with MWCNT Prepared by Solution Blow Spinning. *Polymers* **2022**, *14*, 15.
9. Jalaja, K.; Bhuvaneswari, S.; Ganiga, M.; Divyamol, R.; Anup, S.; Cyriac, J.; George, B.K. Effective SERS detection using a flexible wiping substrate based on electrospun polystyrene nanofibers. *Anal. Methods* **2017**, *9*, 3998-4003.
10. Zhang, H.; Wang, R.; Li, P.; Jia, L.N.; Wang, F.; Liu, Y.B.; Wang, H.; Yu, L.; Li, B. One-Step, Large-Scale Blow Spinning to Fabricate Ultralight, Fibrous Sorbents with Ultrahigh Oil Adsorption Capacity. *ACS Appl. Mater. Interfaces* **2021**, *13*, 6631-6641.
11. Wu, H.J.; Fan, J.T.; Qin, X.H.; Zhang, G. Thermal radiative properties of electrospun superfine fibrous PVA films. *Mater. Lett.* **2008**, *62*, 828-831.
12. Natarelli, C.V.L.; de Barros, H.E.A.; Freitas, H.R.; Bufalo, T.C.E.; Dias, E.S.; de Oliveira, J.E.; Marconcini, J.M. PVA/zein nanofibers obtained by solution blow spinning. *J. Mater. Sci.* **2023**, *58*, 13518-13529.
13. Akduman, C. Preparation and comparison of electrospun PEO/PTFE and PVA/PTFE nanofiber membranes for syringe filters. *J. Appl. Polym. Sci.* **2023**, *140*, 14.
14. Oliveira, J.E.; Mattoso, L.H.C.; Orts, W.J.; Medeiros, E.S. Structural and Morphological Characterization of Micro and Nanofibers Produced by Electrospinning and Solution Blow Spinning: A Comparative Study. *Adv. Mater. Sci. Eng.* **2013**, *2013*, 14.
15. Surendhiran, D.; Cui, H.Y.; Lin, L. Encapsulation of Phlorotannin in Alginate/PEO blended nanofibers to preserve chicken meat from <i>Salmonella</i> contaminations. *Food Packaging Shelf Life* **2019**, *21*, 9.
16. Chen, H.; Chen, X.H.; Chen, H.Y.; Liu, X.; Li, J.X.; Luo, J.; He, A.H.; Han, C.C.; Liu, Y.; Xu, S.S. Molecular Interaction, Chain Conformation, and Rheological Modification during Electrospinning of Hyaluronic Acid Aqueous Solution. *Membranes* **2020**, *10*, 13.
17. Varnaite-Zuravliova, S.; Savest, N.; Baltusnikaite-Guzaitiene, J.; Abraitiene, A.; Krumme, A. The Investigation of the Production of Salt-Added Polyethylene Oxide/Chitosan Nanofibers. *Materials* **2024**, *17*, 17.
18. Jia, J.J.; Lin, Z.H.; Zhu, J.L.; Liu, Y.J.; Hu, Y.L.; Fang, K.J. Anti-adhesive and antibacterial chitosan/PEO nanofiber dressings with high breathability for promoting wound healing. *Int. J. Biol. Macromol.* **2024**, *261*, 13.
